# Glucosamine impedes transforming growth factor β1-mediated corneal fibroblast differentiation by targeting Krüppel-like factor 4

**DOI:** 10.1186/s12929-019-0566-1

**Published:** 2019-10-09

**Authors:** Ying-Jen Chen, Shih-Ming Huang, Ming-Cheng Tai, Jiann-Torng Chen, Chang-Min Liang

**Affiliations:** 1 Graduate Institute of Medical Sciences, National Defense Medical Center, Taipei, Taiwan, Republic of China; 2 Department of Ophthalmology, Tri-Service General Hospital; and School of Medicine, National Defense Medical Center, Number 325, Section 2, Chang-gong Rd, Nei-Hu District, 114 Taipei, Taiwan, Republic of China; 3 Department of Biochemistry, National Defense Medical Center, Taipei, Taiwan, Republic of China

**Keywords:** Glucosamine, Corneal fibroblast, Krüppel-like factor 4

## Abstract

**Background:**

Transforming growth factor (TGF) family members play important roles in the regulation of corneal integrity, and the pathogenesis of corneal fibrosis. Currently, there are no effective agents targeting TGF-β signaling to diminish corneal fibrosis. Glucosamine (GlcN), which is widely used in the treatment of osteoarthritis, abrogates the morphologic effects of TGF-β2 on retinal pigmented epithelial cells in a mouse disease model. Here, we sought to determine whether GlcN would exert beneficial effects against TGF-β1-induced corneal fibrosis.

**Methods:**

In human corneal fibroblasts (HCFs) treated with GlcN, the expression of Krüppel-like factor 4 (KLF4) and its downstream signaling effects were determined in the presence and absence of TGF-β1 using immunoblot analysis. We further explored GlcN inhibition of fibroblast-to-myofibroblast differentiation via KLF4 siRNA. The effect of cycloheximide on KLF4 protein levels with or without GlcN administration was assessed to determine whether GlcN affects the stability of the KLF4 protein.

**Results:**

In HCFs, GlcN induced the expression of KLF4, which regulated the maturation and maintenance of the ocular surface. GlcN partially suppressed the TGF-β1-induced expression of alpha-smooth muscle actin (α-SMA) and reduced the collagen contraction capacity in HCFs, suggesting a decrease in fibroblast-to-myofibroblast differentiation. This effect appeared to be mediated through suppression of Smad2 phosphorylation and ERK-dependent signaling. The levels of KLF4 mRNA were increased by GlcN and decreased by TGF-β1 and the TGF-β1-induced α-SMA mRNA expression was upregulated when the KLF4 gene was silenced. GlcN also appeared to stabilize the KLF4 protein, reducing its turnover in corneal fibroblasts.

**Conclusion:**

These findings shed light on a novel mechanism by which GlcN suppresses TGF-β1-induced fibroblast-to-myofibroblast differentiation through the upregulation of KLF4 expression. Current strategies for treating corneal fibrosis were not effective. Elevating KLF4 levels through the use of GlcN might provide an effective alternative to alleviate the development and progression of corneal fibrosis.

## Background

The development of tissue fibrosis is common to many chronic diseases. Unregulated or persistent fibrogenesis may lead to structural and functional changes in organs that significantly increase the risk of morbidity and mortality. Current evidence indicates that the TGF-β-induced activation of interstitial fibroblasts, myofibroblasts, and renal tubule epithelial cells contributes to the pathological process of fibrosis [1]. TGF-β binding to its receptor (TGF-βR1 or TGF-βR2) leads to activation of Smad and non-Smad signaling pathways, including the extracellular signal-regulated kinase (ERK), JNK, and p38 mitogen-activated protein kinases (MAPK) pathways [2]. The fibrogenic effect of TGF-βR signaling has been described in the kidney, heart, liver, and cornea [3, 4]. Although TGF family members also exert fibrogenic effects in the cornea that can adversely affect the regulation of corneal integrity, no promising TGF-β blockers or other therapeutic agents capable of diminishing corneal fibrosis without inducing adverse effects have been reported [5].

Krüppel-like factor 4 (KLF4) is a member of the zinc-finger class of transcriptional regulators and is required for reprogramming adult fibroblasts into induced pluripotent stem cells [6]. KLF4 interacts with GC-rich or CACCC elements, also called TGF-β1 control elements, in target genes to regulate TGF-β1-induced cell proliferation and differentiation [7]. Given that KLF4 can interact with p300 histone acetyltransferase to activate gene transcription, it is possible that KLF4 also affects histone acetylation via the recruitment of p300 [8]. In addition, KLF4 directly interacts with Smad3 to inhibit myofibroblast differentiation [9]. Results from a serial gene expression analysis revealed that KLF4 is one of the most highly expressed transcription factors in the mouse cornea [10], and the ablation of KLF4 in ocular tissues disrupts corneal epithelial barrier function, which lead to stromal edema [11]. Disrupting KLF4 in KLF4 conditional null mice was shown to induce cytokine cascades that lead to the development of a proinflammatory environment [12]. However, comparatively little is known about the gene network or the regulators involved in the disease pathology underlying corneal fibrosis.

The amino sugar glucosamine (GlcN) is a precursor in the biochemical synthesis of glycosylated proteins and lipids and exhibits both antioxidant and anti-inflammatory effects. GlcN is widely used in the treatment of osteoarthritis (OA) due to its ability to stimulate chondrocyte metabolism*. O*-GlcNAcylation is a noncanonical glycosylation mechanism through which a single *O*-linked *N*-acetylglucosamine (*O*-GlcNAc) moiety is attached to the serine or threonine residue of cellular proteins. *O*-GlcNAcylation is highly sensitive to changes in the cellular environment and interacts extensively with other posttranslational modifications, including those involved in phosphorylation, ubiquitination, acetylation, and methylation [13]. We previously found that GlcN treatment increases cell survival and reduces apoptosis in retinal ganglion cells under oxidative stress and in rat retina subjected to ischemia-reperfusion injury [14]. In the retinal pigment epithelium, GlcN inhibits ICAM-1 expression, modulates *O*-linked glycosylation of the factors involved in NF-κB signaling, and reduces *N*-linked glycosylation of TNF-α-induced ICAM-1 [15]. In addition, in a mouse model of proliferative vitreoretinopathy, GlcN abrogated the morphologic effects of TGF-β2 on retinal pigmented epithelial cells [16].

Although the findings summarized above illustrate the importance of GlcN and KLF4 in the pathogenesis of fibrosis, the underlying mechanisms regulating signaling in the TGF-β pathway during the pathogenic process of corneal fibrosis remain unclear. In an effort to provide new insight into the clinical application of GlcN for corneal fibrosis, we examine whether the effect of GlcN on TGF-β1-induced corneal fibrosis is mediated by KLF4 in human corneal fibroblasts (HCFs).

## Materials and methods

### Ethics statement

All protocols in this study were approved by the Institutional Review Board (IRB) of Tri-Service General Hospital, Taipei, Taiwan, ROC. All methods were performed in accordance with the relevant guidelines and regulations.

### Cell culture and reagents

HCFs from the residual parts of human corneal rims were cultured in Dulbecco’s modified Eagle’s medium (DMEM) at 37 °C under a humidified atmosphere containing 5% CO_2_. HCFs were obtained as follows: After washing the corneas with phosphate-buffered saline (PBS) and treating them with antibiotics, we eliminated the endothelial and epithelial layers, cut the cornea into pieces and incubated the pieces in collagenase-containing DMEM supplemented with 10% fetal bovine serum (FBS) to dissociate HCFs.

Anti-KLF4 and anti-fibronectin antibodies were purchased from Abcam (Cambridge, United Kingdom). Anti-α-actinin, anti-collagen type 1, anti-β-actin, anti-Smad7, anti- TGF-βR2, and anti-phospho-TGF-βR2 antibodies were purchased from Santa Cruz Biotechnology (Santa Cruz, CA, USA). Anti-Smad2, anti-phospho-Smad2, anti-Smad3, and anti-phospho-Smad3 antibodies were purchased from Cell Signaling Technology (Beverly, MA, USA). Anti-α-SMA antibody was purchased from Sigma Chemical Co. (St. Louis, MO, USA).

### Real-time quantitative reverse transcription PCR

After the isolation of total RNA, real-time reverse-transcription PCR was carried out using a StepOne™ Real-Time PCR system according to the manufacturer’s instructions. The following primer pairs were used: GAPDH, forward: 5′-CTTCATTGACCTCAACTAC-3′ and reverse: 5′-GCCATCCACAGTCTTCTG-3′; α-SMA, forward: 5′-CTATGAGGGCTATGCCTTGCC-3′ and reverse: 5′-GCTCAGCAGTAGTAACGAAGGA-3′; KLF4, forward: 5′-CAAGCCAAAGAGGGGAAGAC-3′ and reverse: 5′-CGTCCCAGTCACAGTGGTAA-3′; COL1A1, forward: 5′-GAGGGCCAAGACGAAGACATC-3′ and reverse: 5′-CAGATCACGTCATCGCACAAC-3′; fibronectin, forward: 5′-GAGAATAAGCTGTACCATCGCAA-3′ and reverse: 5′-CGACCACATAGGAAGTCCCAG-3′;.

### Western blot analysis

After washing with PBS, the target cells were harvested in RIPA lysis buffer containing protease inhibitors. Quantified protein samples were separated using sodium dodecyl sulfate-polyacrylamide gel electrophoresis and electrotransferred to PVDF membranes. The membranes were washed with Tris-buffered saline and incubated overnight at 4 °C with primary antibodies, followed by the application of a horseradish peroxidase-conjugated secondary antibody (Jackson ImmunoResearch Laboratories, West Grove, PA, USA). The protein contents were then quantified using the standard enhanced-chemiluminescence procedure. The results from the independent experiments conducted with Western blot scanning densitometry were calculated using ImageJ software.

### Immunofluorescence

HCFs were cultured on 4-well cell culture slides (30,104; SPL Life Sciences, Pocheon, Korea). After fixation in 4% paraformaldehyde, the cells were incubated with 0.1% Triton X-100 in PBS for 30 min, blocked for 1 h with 1% bovine serum albumin in PBS, and incubated with rabbit anti-KLF4 (Abcam, Cambridge, UK) and rabbit anti- α-SMA (Sigma Chemical Co, MO, USA) antibodies. The secondary antibodies were anti-rabbit IgG and anti-mouse IgG (BioLegend, CA, USA). Nuclei were counterstained with 4′,6-diamidino-2-phenylindole dihydrochloride (DAPI) (Sigma Chemical Co). Fluorescence images were obtained using an Olympus CKX41 microscope (Tokyo, Japan).

### Collagen contraction assay

HCFs were harvested and then resuspended in DMEM at a density of 1.1 × 10^7^ cells/ml. Aliquots of the suspension were then added to a mixture of reconstitution buffer containing type I collagen (Nitta Gelatin, Osaka, Japan) with or without GlcN and/or TGF-β1 (10 ng/ml). Once the gels had polymerized and released from the sides of the wells, time-dependent changes in gel size were assessed using ImageJ software.

### Transfection of siRNA targeting KLF4

All siRNAs were synthesized by Dharmacon Research (Lafayette, CO, USA). HCFs were transfected with KLF4 siRNA or siControl (siGENOME SMARTpool, Dharmacon) using the DharmaFECT 1 transfection reagent. The siRNA strands were deprotected as instructed by the manufacturer.

### Statistical analyses

All data are presented as the mean ± standard deviation obtained from three independent experiments. Statistical differences between groups were assessed using Student’s t test or analysis of variance (ANOVA). Values of *p* < 0.05 were considered significant.

## Results

### GlcN increases KLF4 gene and protein expression

**Fig. 1:**
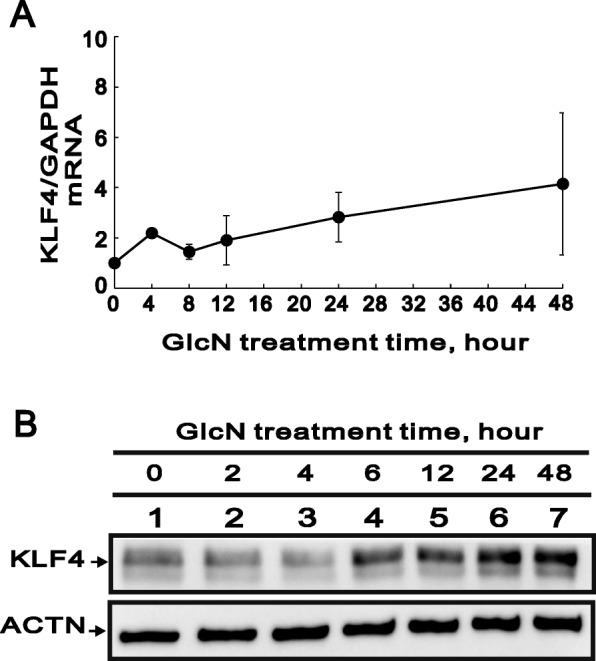
Effects of GlcN on KLF4 mRNA and protein levels in HCFs. (**a**) Real-time PCR analysis of relative KLF4 mRNA levels in HCFs after application of GlcN (5 mM) for the indicated times. KLF4 mRNA levels were normalized to those of GAPDH mRNA, which served as a loading control. Symbols depict the mean ± SD (*n* = 3). (**b**) Immunoblot analysis showing the levels of KLF4 protein at the indicated GlcN treatment times. ACTN served as a loading control

To determine the optimal time for GlcN treatment, HCFs were treated with 5 mM GlcN for 0, 4, 8, 12, 24, or 48 h. The expression level of KLF4 mRNA was increased after 8 h of GlcN treatment, and a substantially greater increase in KLF4 mRNA expression level was detected after 48 h of GlcN treatment (Fig. 1a). Similarly, the results from the western blot analysis showed that the levels of KLF4 protein were increased after 6, 12, 24, and 48 h of treatment, with the highest levels found at 48 h (Fig. 1b). These results show that GlcN promotes the elevated expression of KLF4 mRNA and protein in HCFs.

### GlcN suppresses TGF-β1-induced α-SMA expression via KLF4 upregulation

**Fig. 2:**
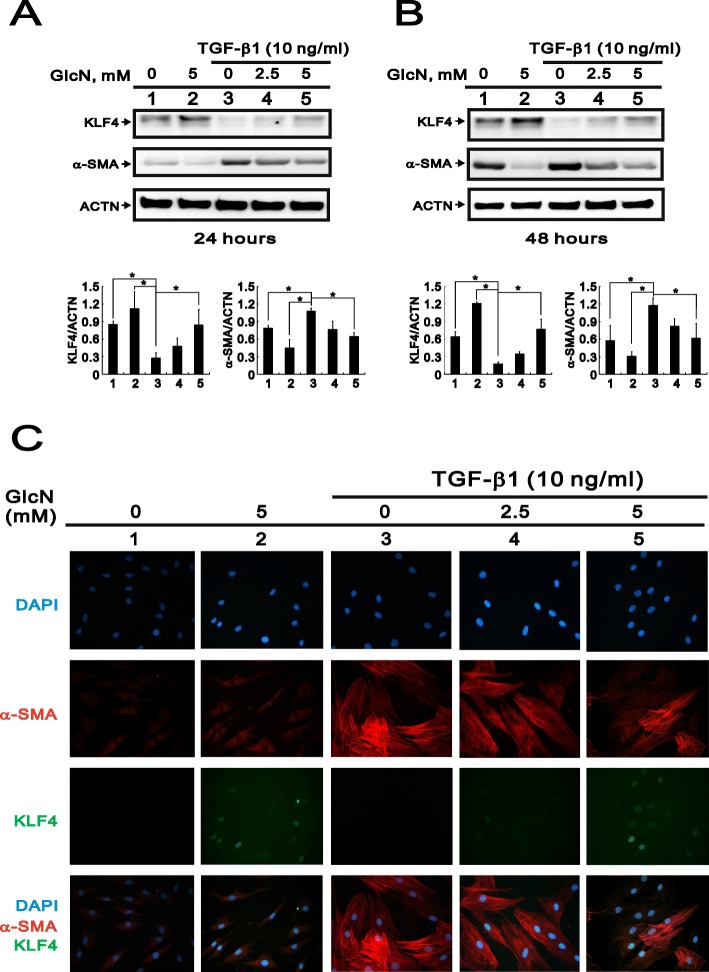
Effects of GlcN on TGF-β1-induced α-SMA expression in HCFs. (**a** and **b**) Immunoblots showing the expression levels of KLF4 and α-SMA in HCFs after application of the indicated concentration of GlcN for 24 h (**a**) or 48 h (**b**), with or without 10 ng/ml TGF-β1. ACTN served as a loading control. Asterisk indicated a statistically significant difference (*p* < 0.05) between groups. Each value represented the mean ± SD for three experiments. (**c**) Photomicrographs showing the immunofluorescent staining of KLF4 and α-SMA in HCFs after application of the indicated concentration of GlcN for 24 h, with or without 10 ng/ml TGF-β1

It was previously reported that KLF4 expression decreases fibrotic responses in renal tubular epithelial cells during diabetic nephropathy [17]. Therefore, we assessed the effect of GlcN on the differentiation of HCFs into myofibroblasts in the TGF-β1 induced model. First, we observed that TGF-β1 significantly suppressed KLF4 protein expression and induced α-SMA protein expression at 24 h and 48 h treatment using the western blotting analysis (Fig. 2a and b, compare lane 3 with lane 1). Immunoblots showed that after 24 or 48 h of treatment with GlcN, the levels of α-SMA expression were reduced in the presence or absence of TGF-β1, which was accompanied by a corresponding upregulation of KLF4 expression (Fig. 2a and b). These effects were further confirmed by immunofluorescence staining, which revealed that GlcN treatment markedly suppressed TGF-β1-induced α-SMA expression, while increasing nuclear levels of KLF4 protein colocalized with the nuclear signals of DAPI (Fig. 2c). Our findings suggest that GlcN might inhibit TGF-β1-induced HCF differentiation into myofibroblasts through the upregulation of KLF4.

### GlcN suppresses the expression of type I collagen by TGF-β1 induction

**Fig. 3:**
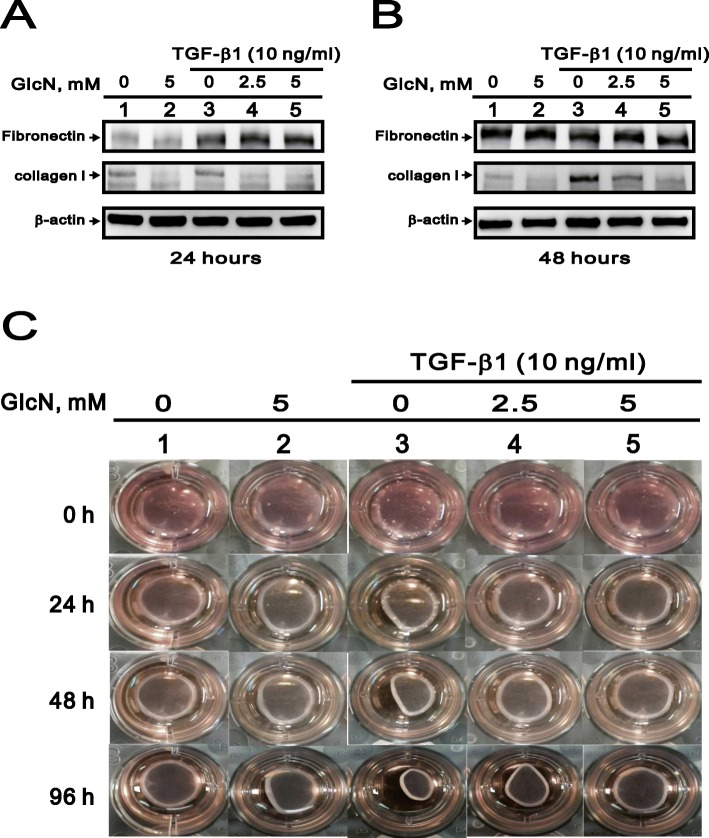
Effect of GlcN on collagen contraction capacity in HCFs. (**a** and **b**) Immunoblots showing the expression levels of fibronectin and collagen 1 in HCFs after application of the indicated concentration of GlcN for 24 h (**a**) or 48 h (**b**), with or without 10 ng/ml TGF-β1. β-actin served as a loading control. (**c**) Collagen contraction analysis after application of the indicated concentration of GlcN for the indicated times

The development of fibrosis was characterized by the enhanced production of extracellular matrix (ECM) [18]. To elucidate the effect of GlcN on production of the ECM, we assessed its effect on the production of fibronectin and type I collagen (collagen I) by HCFs in the presence and absence of TGF-β1. As shown in Fig. 3a and b, GlcN only significantly blunted TGF-β1-induced collagen I, not fibronectin, production at 24 h and 48 h treatments. Using collagen-based cell contraction assays, we also observed that GlcN ameliorated TGF-β1 induced cell contraction (Fig. 3c). After 48 h and 96 h of exposure, TGF-β1 increased the collagen contraction capacity within cells, but this effect was substantially inhibited by administration of 2.5 mM or 5.0 mM GlcN. These results indicate that GlcN significantly suppressed TGF-β1-induced ECM production and collagen contraction capacity, possibly through the inhibition of collagen I expression.

### The effect of GlcN on Smad-dependent and Smad-independent signaling in TGF-β signaling pathway

**Fig. 4:**
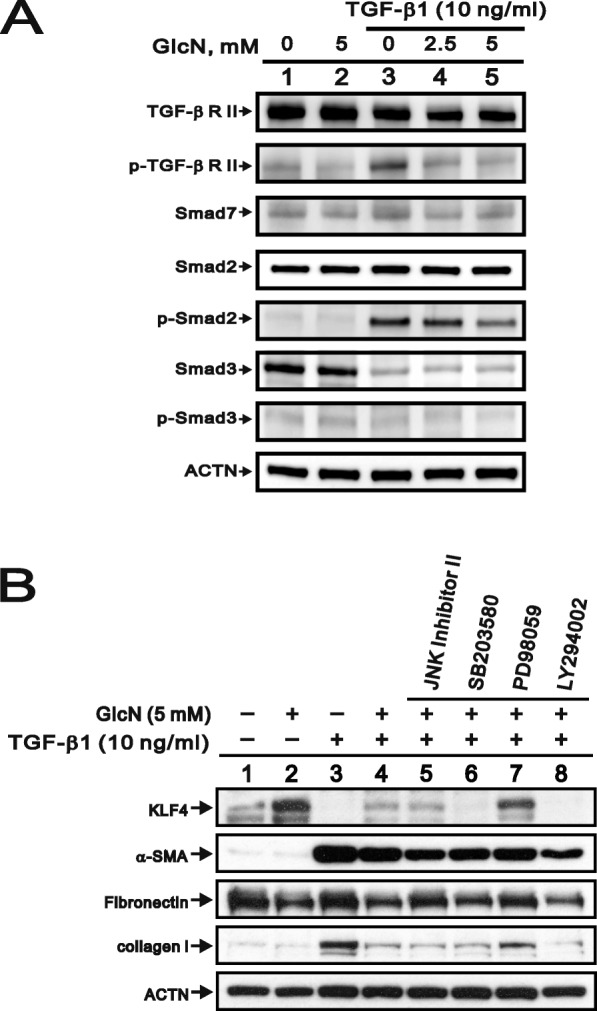
Effects of GlcN on Smad-dependent or -independent signaling in HCFs. (**a**) Immunoblot analysis for detection of total and phosphorylated TGF-βRII, Smad7, total and phosphorylated Smad2, and total and phosphorylated Smad3 in HCFs. GlcN was applied for 24 h, with or without 10 ng/ml TGF-β1. ACTN served as a loading control. (**b**) Immunoblot analysis for detection of KLF4, α-SMA, fibronectin, and collagen I in HCFs. GlcN (5 mM) and/or TGF-β1 (10 ng/ml) were applied for 24 h where indicated (+), with or without indicated signaling inhibitor. ACTN served as a loading control)

Because the TGF-β signaling pathway plays a key role in the pathophysiology of tissue fibrogenesis, we sought to determine whether GlcN affects signaling via TGF-β receptors (TGF-βRs) and the canonical Smad-dependent pathway in the HCFs. As shown in Fig. 4a, GlcN inhibited the TGF-β1-mediated phosphorylation of TGF-βR2 and Smad2 in a dose-dependently manner, though GlcN had no effect on Smad7 expression, with or without TGF-β1. Smad3 protein expression was abrogated in HCFs stimulated by TGF-β1, therefore, it was difficult to evaluate the effect of GlcN on Smad3 phosphorylation levels (Fig. 4a, compare lanes 3–5 with lane 1).

In addition to the Smad-dependent pathway, TGF-β1 might mediate the many Smad-independent pathways, including JNK, ERK, p38, and PI3K, to exert its signal transduction [19, 20]. Here, we used specific kinase inhibitors for the abovementioned pathways to address the working mechanisms of TGF-β1 and GlcN. GlcN did increase KLF4 protein and decrease α-SMA protein and TGF-β1 did increase α-SMA and collagen I proteins and decrease KLF4 protein in HCFs (Fig. 4b, compare lane 2 or 3 with lane 1). In a group of GlcN and TGF-β1 co-treatment, GlcN consistently suppressed the effects of TGF-β1 on the expression levels of the KLF4, α-SMA, and collagen I proteins (Fig. 4b, compare lanes 3–4). There was no apparent effect of GlcN and TGF-β1 on the expression level of fibronectin. Compared with the expression levels in the group of cells co-treated with GlcN and TGF-β1 (lane 4 of Fig. 4b), pretreating HCFs with the ERK1/2 inhibitor PD98059 (50 μM) increased KLF4 and collagen I protein expression, whereas pretreating the cells with the p38 MAPK inhibitor SB203580 (30 μM) or PI3K/Akt inhibitor LY294002 (30 μM) suppressed KLF4 protein expression (Fig. 4b, compare lanes 6–8 with lane 4). None of four inhibitors had an apparent effect on the expression of the α-SMA and fibronectin proteins.

### Effect of KLF4 knockdown on α-SMA, fibronectin, and collagen I expression with or without TGF-β1 and GlcN

**Fig. 5:**
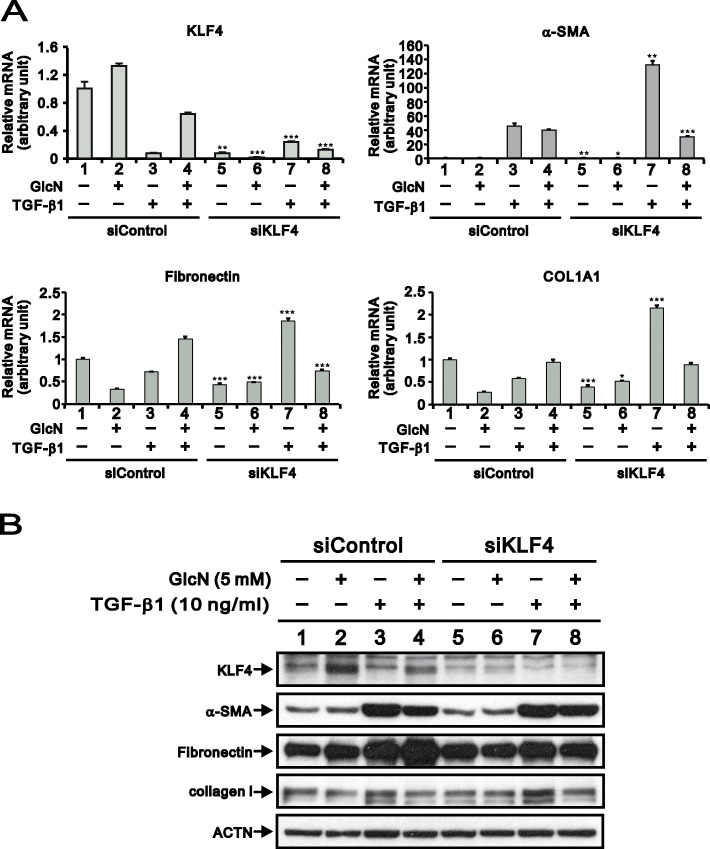
Effects of GlcN-induced KLF4 mRNA and protein expression on corneal fibrosis in HCFs. (**a**) Real-time PCR analysis showing the expression levels KLF4, α-SMA, fibronectin, and COL1A1 mRNA in HCFs transfected with siControl or siKLF4. GAPDH served as a loading control. GlcN (5 mM) and/or TGF-β1 (10 ng/ml) were applied for 24 h where indicated (+). Bars depict the mean ± SD (*n* = 3). *p < 0.05, **p < 0.01, ***p < 0.001. (**b**) Immunoblot analysis for detection of KLF4, α-SMA, fibronectin, and collagen I in HCFs transfected with siControl or siKLF4. GlcN and/or TGF-β1 were applied as indicated. ACTN served as a loading control

As shown in Figs. 1 and 2, GlcN promotes KLF4 gene and protein expression while decreasing both α-SMA levels and ECM accumulation. We further explored the GlcN inhibition of fibroblast-to-myofibroblast differentiation via KLF4 using a silencing strategy. HCFs were transfected with KLF4 siRNA to knock down the endogenous KLF4 mRNA expression, which real-time PCR analysis confirmed a silencing efficacy of > 90% (Fig. 5a, KLF4, compare histograms 1 and 5). GlcN increased KLF4 mRNA expression. TGF-β1 had the opposite effect, decreasing KLF4 mRNA expression, but this effect could be reversed by GlcN (Fig. 5a, KLF4, compare histograms 1–4). Indeed, there was a dramatic induction of α-SMA mRNA expression by TGF-β1 in siControl group which was further enhanced in the siKLF4 group (Fig. 5a, α-SMA, compare histograms 3 and 7 with histogram 1). TGF-β1-induced α-SMA mRNA expression was suppressed by GlcN in the siControl group and siKLF4 group. Also decreasing in the siKLF4 group as α-SMA, was expression of fibronectin and collagen I (Fig. 5a, α-SMA, fibronectin and collagen I, compare histograms 1 and 5). GlcN enhanced the stimulatory effect of TGF-β1 on fibronectin and collagen I expression in the siControl group (Fig. 5a, fibronectin and collagen I, compare histograms 3 and 4), whereas no induction by TGF-β1 on fibronectin and collagen I. TGF-β1 had enhancing effect on levels of fibronectin or collagen I mRNA in the siKLF4 group (Fig. 5a, fibronectin and collagen I, compare histograms 5–8). GlcN still had suppressive effect on the TGF-β1-induced fibronectin or collagen I mRNA in the siKLF4 group (Fig. 5a, fibronectin and collagen I, compare histograms 7 and 8).

To confirm the effects of the downregulated KLF4 on α-SMA, fibronectin, and collagen I mRNA levels in HCFs, we used Western blotting analysis to assess the effects of downregulated KLF4 on the levels of α-SMA, fibronectin, and collagen I proteins (Fig. 5b). TGF-β-induced α-SMA protein expression was observed in both the siControl and siKLF4 groups (Fig. 5b, compare lane 3 and lane 1 or lane 7 and lane 5). Knockdown of KLF4 by siRNA partially rescued the inhibition of GlcN on the TGF-β1-induced α-SMA protein (Fig. 5b, compare lanes 4 and 8). However, the current silencing strategy apparently affected the response of fibronectin and collagen I to TGF-β1, GlcN, and both TGF-β1 and GlcN in combinations. The inconsistent findings remain to be addressed in the future.

### GlcN increases the stability of the KLF4 protein

**Fig. 6:**
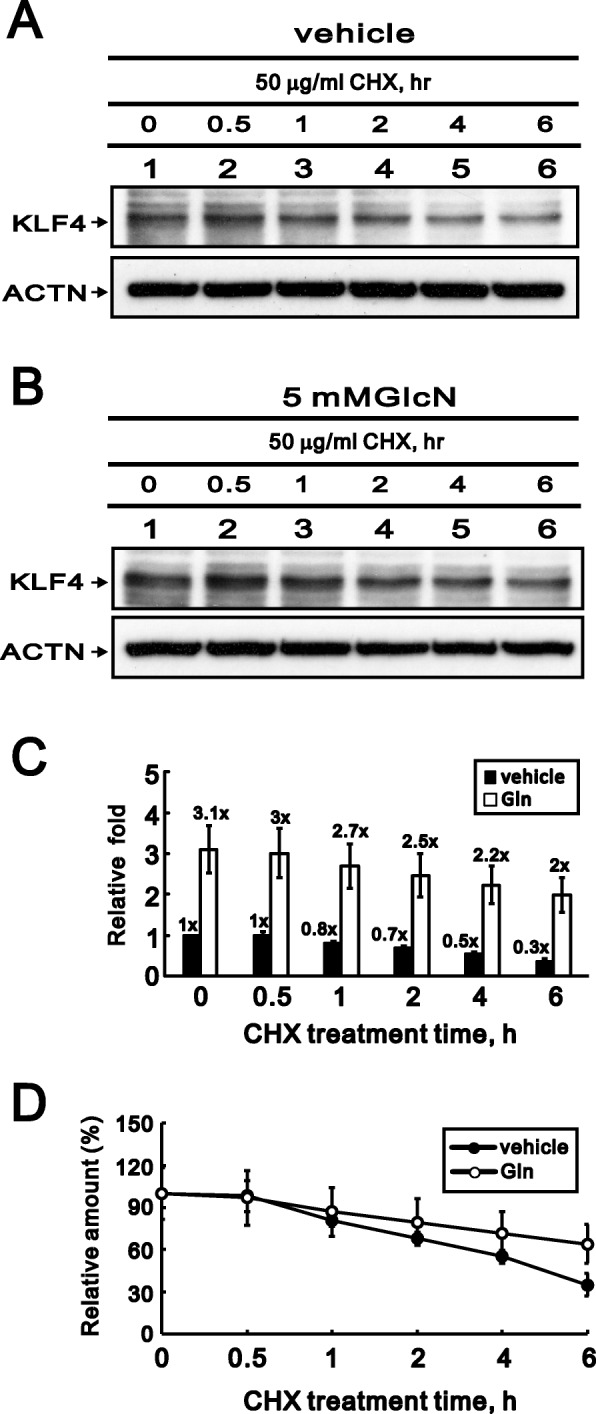
Effects of GlcN on the stability of KLF4 protein in HCFs. (**a** and **b**) Immunoblots showing levels of KLF4 in HCFs after treatment with vehicle (**a**) or 5 mM GlcN (**b**) plus 50 ng/ml CHX for the indicated times. ACTN served as a loading control. (**c** and **d**) Graphic representations of KLF4 protein levels derived from western analyses like those depicted in panels A and B. Shown are the relative fold and percentage changes in the presence of CHX. Bars and symbols depict the mean ± SD (*n* = 3)

An earlier study showed that KLF4 mediates the link between TGF-β1-induced activation of gene transcription and H3 acetylation [21]. To determine whether GlcN affects the stability of KLF4 protein, we assessed the effect of cycloheximide (CHX), a de novo protein synthesis inhibitor, on KLF4 protein levels at different time points with or without GlcN administration (Fig. 6a and b). In HCFs treated with vehicle or 5 mM GlcN, the levels of KLF4 protein were markedly decreased after being treated for 1 h with 50 μg/ml CHX (Fig. 6c). After 6 h, however, there was a 0.3- to 1-fold decrease in KLF4 levels in the vehicle group but a 2.0- to 3.1-fold decrease in KLF4 levels in the 5 mM GlcN group. The average residual amount of KLF4 protein was more stable in the 5 mM GlcN group than the vehicle group (Fig. 6d). The addition of GlcN reduced the rate of decline in the level of KLF4 protein in the presence of CHX, which suggests that GlcN increases the stability of the KLF4 protein by reducing its turnover rate.

## Discussion

Our data provide evidence that GlcN reduces TGF-β1-induced fibroblast-to-myofibroblast differentiation by inhibiting the phosphorylation of Smad2, ERK-dependent signaling, and increasing KLF4 expression. Levels of KLF4 mRNA and protein were increased by GlcN and decreased by TGF-β1. TGF-β1-induced α-SMA mRNA expression was upregulated when KLF4 gene was silenced. GlcN also appears to stabilize the KLF4 protein, reducing its turnover in corneal fibroblasts. Importantly, these findings fill a gap in our knowledge about the potentially suppressive effect of GlcN on corneal fibrogenesis mediated by TGF-β1 and the mechanism underlying this effect.

In line with our observation that GlcN decreases TGF-β1-induced Smad2 phosphorylation in HCFs, it was previously reported that GlcN markedly reduces elevated Smad2 phosphorylation in human renal epithelial cells to attenuate TGF-β signaling [22]. We also observed that GlcN reduces TGF-β1-induced phosphorylation of its receptor, TGF-βR2, in a dose-dependent manner. In recent years, considerable concern has arisen over the crucial involvement of TGF-βR2 in systemic inflammation and fibrosis, including fibrosis of lung, bladder, skin, and kidney. TGF-βR2 signaling via TGF-β/Smad3 or NF-κB in kidney fibroblasts or tubular epithelial cells exerts diverse effects during renal fibrosis and inflammation [23]. In a healing rat cornea following post-alkali burn, blocking TGF-βR2 overtly suppresses scarring and neovascularization [23]. GlcN may mediate posttranslational modification of TGF-βR2, specifically via N-linked glycosylation, to alter the cellular sensitivity to TGF-β [24]. In a study of the inhibitory effect of GlcN on renal fibrosis, it was found that GlcN inhibits N-linked glycosylation of TGF-βR2 and contributed to the disruption of TGF-βR2 trafficking [22]. These results raise the possibility that regulation of TGF-βR2 glycosylation by GlcN may be involved in several posttranslational modifications.

In rat primary skeletal muscle cells, GlcN reportedly increases the levels of the activated (phosphorylated) form of the phosphatase PTEN [25]. During vascular smooth muscle cell differentiation, phosphorylated PTEN loses its ability to dephosphorylate KLF4, and the phosphorylated KLF4 enhances p300 histone acetyltransferase activity, which can modify histone acetylation and, in turn, gene expression [21]. Another possible explanation for the underlying molecular mechanisms linking GlcN and KLF4 involves the posttranslational modifications of O-linked N-acetylglucosamine (*O*-GlcNAc). *O*-GlcNAc is linked to serine or threonine residues in proteins through a reaction catalyzed by O-linked-β-N-acetylglucosaminyl-transferase (OGT) [26]. To catalyze these posttranslational modifications, OGT interacts with a histone deacetylase complex by binding to the corepressor mSin3A [27]. Notably, GlcN increases *O*-GlcNAc levels in a dose-dependent manner [14], and the posttranslational modification of the mammalian proteasome by *O*-GlcNAc can inhibit its proteolytic function [28]. In human prostate cancer cells, proteasomal chymotrypsin-like activity is inhibited by GlcN in a dose-dependent manner, which highlights GlcN as an important negative regulator of proteasomal activity in pathological processes. Taken together, these studies suggest that this *O*-GlcNAc modification might be the mechanism by which the stability of KLF4 protein is increased by GlcN in the present study.

TGF-β1 might mediate through the Smad-dependent and Smad-independent pathways, including JNK, ERK, p38, and PI3K, to exert its physiological functions [19, 20]. Consistent with previous evidences, inactivation of the ERK signaling pathway substantially diminishes the TGF-β2-induced upregulation of ECM components [29]. The PI3K/Akt pathway is involved in transducing the TGF-β2 signal to induce type I collagen synthesis [30]. Recent studies support that *O*-GlcNAcylation has extensive cross talk with phosphorylation to modulate signaling, transcription, translation, and cytoskeletal functions [31–33]. In conjunction with *O*-GlcNAc modification of nuclear proteins, such as Sp1, GlcN might alter their transactivation activity, turnover, or protein-protein interaction. KLF4 activity had been shown to be negatively regulated by ERK1 and ERK2 phosphorylation at the post-translational level [34, 35]. KLF4 has been demonstrated that it is degraded in response to TGF-β signaling [36]. We failed to elucidate the cross-talk between GlcN and TGF-β1 via four specific kinase inhibitors, suggesting that complicated cross-talk among these signaling pathways. This issue remains to be further investigated in the future.

KLF4 is a well-known repressor involved into the regulation of TGF-β1-induced smooth muscle actin gene expression [37], while under different conditions it may play a completely opposite role [35]. Our current Fig. 5 data showed that downregulation of KLF4 not only decreased α-SMA mRNA, but also fibronectin and collagen I mRNAs, suggesting that KLF4 might be an activator in the TGF-β1-induced gene network. However, the effect of GlcN on the induction of α-SMA gene and protein expression by TGF-β1 inconsistent. In eukaryotes, transcription, translation and degradation are often tortuously coupled with each other through feedback loops to modulate the protein expression [38]. Hence, our research raises this important issue to address the regulatory mechanism of KLF4 for α-SMA expression in transcription and translation levels.

## Conclusions

In summary, our findings suggest a novel mechanism by which GlcN causes repression of TGF-β1-induced fibroblast-to-myofibroblast differentiation through the upregulation of KLF4. Current strategies for treating corneal fibrosis are not effective. It is expected that treatments aimed at elevating KLF4 may offer an alternative to diminish the fibrotic corneal response by suppressing fibroblast-to-myofibroblast differentiation. Although we investigate several aspects of action of GlcN during fibrotic processes, the precise mechanism underlying the interplay between GlcN and KLF4 in transcription, translation, and post-translation stages has yet to be well defined. More research is still needed to expand our understanding of corneal fibrosis.

## Data Availability

All supporting data have been shown in current manuscript.

## Abbreviations

CHX: cycloheximide
DMEM: Dulbecco’s modified Eagle’s medium
ERK: extracellular signal-regulated kinase
FBS: fetal bovine serum
GlcN: glucosamine
HCFs: human corneal fibroblasts
KLF4: Krüppel-like factor 4
MAPK: mitogen-activated protein kinase
OA: osteoarthritis
*O*-GlcNAc: *O*-linked *N*-acetylglucosamine
PBS: phosphate buffered saline
PCR: polymerase chain reaction
RIPA: radio-immune precipitation assay
siControl: control siRNA
siKLF4: KLF4 siRNA
TGF-β: transforming growth factor beta
α-SMA: alpha smooth muscle actin
